# The evidence for services to avoid or delay residential aged care admission: a systematic review

**DOI:** 10.1186/s12877-019-1210-3

**Published:** 2019-08-08

**Authors:** Julie A. Luker, Anthea Worley, Mandy Stanley, Jeric Uy, Amber M. Watt, Susan L. Hillier

**Affiliations:** 1 Division of Health Sciences, University of South Australia, North Terrace, Adelaide, South Australia Australia; 2 ECH Incorporated, 174 Greenhill Road, Parkside, South Australia Australia; 3 School of Medical and Health Sciences, Edith Cowan University, 270 Joondalup Drive, Joondalup, Western Australia Australia

**Keywords:** Aging in place, Community dwelling, Independent living, Health services, Community, Systematic review

## Abstract

**Background:**

Interventions that enable people to remain in their own home as they age are of interest to stakeholders, yet detailed information on effective interventions is scarce. Our objective was to systematically search and synthesise evidence for the effectiveness of community-based, aged care interventions in delaying or avoiding admission to residential aged care.

**Method:**

Nine databases were searched from January 2000 to February 2018 for English publications. Reference lists of relevant publications were searched. The databases yielded 55,221 citations and 50 citations were gleaned from other sources. Where there was sufficient homogeneity of study design, population, intervention and measures, meta-analyses were performed. Studies were grouped by the type of intervention: complex multifactorial interventions, minimal/single focus interventions, restorative programs, or by the target population (e.g. participants with dementia).

**Results:**

Data from 31 randomised controlled trials (32 articles) that met our inclusion criteria were extracted and analysed. Compared to controls, complex multifactorial interventions in community aged care significantly improved older adults’ ability to remain living at home (risk difference − 0.02; 95% CI -0.03, − 0.00; *p* = 0.04). Commonalities in the 13 studies with complex interventions were the use of comprehensive assessment, regular reviews, case management, care planning, referrals to additional services, individualised interventions, frequent client contact if required, and liaison with General Practitioners. Complex interventions did not have a significantly different effect on mortality.

Single focus interventions did not show a significant effect in reducing residential aged care admissions (risk difference 0, 95% CI -0.01, 0.01; *p* = 0.71), nor for mortality or quality of life.

Subgroup analysis of complex interventions for people with dementia showed significant risk reduction for residential aged care admissions (RD -0.05; 95% CI -0.09, -0.01; *p* = 0.02). Compared to controls, only interventions targeting participants with dementia had a significant effect on improving quality of life (SMD 3.38, 95% CI 3.02, 3.74; *p* < 0.000001).

**Conclusions:**

Where the goal is to avoid residential aged care admission for people with or without dementia, there is evidence for multifactorial, individualised community programs. The evidence suggests these interventions do not result in greater mortality and hence are safe. Minimal, single focus interventions will not achieve the targeted outcomes.

**Trial registration:**

PROSPERO Registration CRD42016050086.

**Electronic supplementary material:**

The online version of this article (10.1186/s12877-019-1210-3) contains supplementary material, which is available to authorized users.

## Background

Interventions and services that enable people to remain living in their own home as they age are of great interest to older people, policy makers and the health and welfare sectors. The majority of older people choose to remain in their own homes for as long as possible, however this is often contingent on access to suitable support that is responsive to their changing needs [1–3].

Improvements in living standards and healthcare have led to people living longer, with increasing proportions of the population aged over 65 years [4, 5]. In the Organisation for Economic Co-operation and Development countries these demographic changes are predicted to at least double the long-term care costs for people aged over 65 years by 2050 [6].

Policy makers and aged care service providers are keen to understand interventions that can ease the pressure on the health and aged care sectors, and reduce the need for long-term residential aged care. In many developed countries there has been a shift from residential care to various models of community-based health and social care for older people. Government policies in many countries now focus on delaying or avoiding the need for long-term residential aged care through the development of person-centred, early intervention and preventative services such as in Australia [7], Sweden [8], New Zealand [9], and England [10].

### Description of the intervention

A wide variety of community-based, aged care interventions have emerged in recent years aimed at supporting people in their homes and delaying or avoiding residential aged care (also known as residential care, nursing home). While all fit a broad category of preventative community aged care, it is challenging to understand the similarities and differences between these interventions as there is no agreed nomenclature, and the elements of the services provided are often poorly described. Very little is known about the effectiveness of any of these interventions.

Previous publications have attempted to categorize the various interventions in community aged care but confusion and overlap remains an issue. Interventions and approaches to care previously described include:

*Centre-based wellness programs.* Wellness has been defined as a multidimensional state of being, describing the existence of positive health in an individual as exemplified by quality of life and a sense of wellbeing [11]. Wellness programs are frequently run from community centres, with transport sometimes provided for participants to attend.

*Re-enablement or restorative home care* has been defined as a time-limited program (typically 6–12 weeks) involving multiple visits to a person’s home by multidisciplinary professionals. It aims to help older people regain functional independence [12]. In this review we will consider falls prevention interventions in this category even though they frequently run longer than 12 weeks.

*Case management* is a complex intervention usually provided by a central worker ([13],p1). The Case Management Society of Australia describes case management as *“a collaborative process of assessment, planning, facilitation and advocacy for options and services to meet an individual’s holistic needs through communication and available resources to promote quality cost-effective outcomes”* [14]. While there is no single definition of case management as practiced within aged community settings, several characteristics of case management in community aged care have been identified including: *“a collaborative process with the family carer; employing a planned approach to achieve client outcomes with cost-efficiency; being based in the community aged care sector”* ([15],p2)*.*

*Consumer directed care* has been defined as “*interventions where consumers were explicitly given choice and/or control of services”* ([13],p3).

*Complex interventions (*e.g. *multifactorial preventative home visits)* is a term used to encapsulate a wide variety of services and complex, multifactorial, individualised interventions aimed at maintaining health and autonomy and preventing disability [16], with case management a key component.

In our analysis of the literature we divided studies into these sub-groupings where possible, but remained open to other sub-groupings that may become evident such as services targeting specific conditions or needs (e.g. dementia specific interventions).

### Previous systematic reviews

Some previous reviews have explored interventions aimed at reducing residential aged care admissions including an earlier systematic review of systematic reviews conducted by Tourigny and colleagues (2015) [17]. These authors concluded that no reviews published prior to 2011 had demonstrated that preventative home visits avoid or delay residential aged care admissions.

An updated systematic review by Mayo-Wilson (2014) on preventative home visiting included evidence published to 2012 [18]. This meta-regression analysis of 26 randomised control trials (RCTs) did not find a significant reduction in the risk of being admitted to an institution by time point, age of participants, type of visitor or number of home visits.

Beswick and colleagues (2008) systematically reviewed RCTs assessing community-based multifactorial interventions for older people living at home and published prior to January 2005. They reported that these complex interventions reduced residential aged care admissions (relative risk 0.87, 95% CI 0.83–0.90), but not death (1.00, 0.97–1.02). The Beswick review did not provide information on the interventions or outcomes in individual studies and was therefore of limited value in informing other end users [19].

### Objectives of this systematic review

No previous systematic review has itemised the elements of the interventions used in included RCTs, thereby limiting their ability to inform service providers and researchers regarding practical approaches to delivering effective care and services.

Therefore this comprehensive systematic review sought to update and synthesise evidence for the effectiveness of community-based, aged care interventions in delaying or avoiding admission to residential aged care for older adults. Secondary objectives were to report the effectiveness of these interventions in maintaining or improving other outcomes such as quality of life and mortality, and where possible, to itemise the elements used in the interventions.

## Methods

This systematic review was registered with the International Prospective Register of Systematic Reviews (PROSPERO) in October 2016 (http://www.crd.york.ac.uk/PROSPERO/display_record.asp?ID=CRD42016050086). We report the review in accordance with the PRISMA Checklist [20].

### Criteria for considering studies for this review

#### Types of studies

Only studies that reported the outcome of ‘remaining home or avoiding residential aged care’ were included. We considered any intervention design including RCTs, controlled trials, cohort studies, or case controlled studies, conducted in any country.

#### Types of participants

Adults 65 years or older, living in their own homes in the community. No restriction was placed on disease or health status.

#### Context

The context of interest was community living including metropolitan or rural communities, retirement homes and independent living units, but excluded residential aged care.

#### Intervention/exposure

Included studies considered any intervention, model, activity, service or program that promotes ‘aging in place’, when the ‘place’ is community (the person’s home). Interventions included those that aimed to prevent or address functional decline, or maintain wellbeing and independence in older adults.

#### Comparator/control

Studies with any or no comparator were considered.

#### Primary outcome

Avoiding residential aged care admission or time remaining at home.

#### Secondary outcomes

Quality of life outcomes, mortality, morbidity or independence measures such as the Barthel Index; Modified Rankine Score; participation levels; health and well-being measures (e.g. SF36); health adjusted QoL (health economic data); healthcare utilization (including hospital admissions); or adverse events.

#### Exclusion criteria

Non-English publications, qualitative studies, and studies on palliative care or end of life care.

### Information sources

Electronic database searches were conducted in MEDLINE, EMBASE, PsycINFO, the Cochrane Database of Systematic Reviews, Cochrane Central Register of Controlled Trials (CENTRAL), Cochrane Methodology Register, AMED, CINAHL and Ageline from January 2000 to February 2018.

### Search

A research librarian helped develop a search string in MEDLINE (see Additional file 1), which was adapted for the other databases. The databases were searched, results were entered into Endnote folders, and any duplicates and irrelevant titles were removed (AW). Reference lists of relevant reviews were hand searched to identify additional potential studies.

### Study selection

**Fig. 1:**
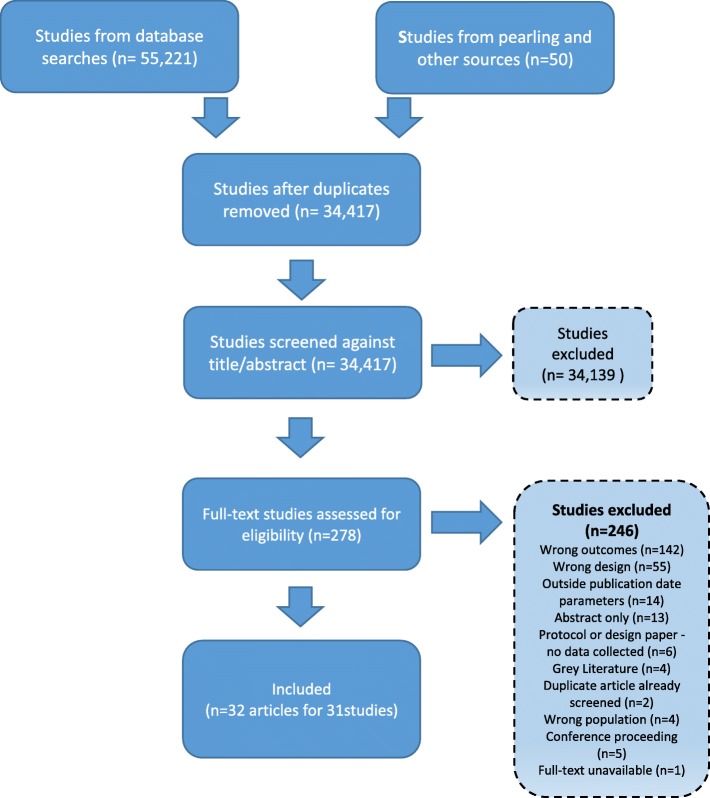
Flow diagram of study selection

Study selection against the review’s criteria was managed within Covidence software (www.covidence.org/). Study selection was conducted in two phases independently by two reviewers (AW, JU, or JL). In the first phase all titles and abstracts were screened and studies were excluded if both reviewers agreed to exclude. Title/abstracts without consensus agreement underwent full-text screening. In the second phase of full-text screening, consensus was reached to include or exclude studies from the review and differences were resolved through discussion or by another author (SH). The reasons for full-text exclusion were recorded and the selection process was mapped in a PRISMA flow chart (Fig. 1).

### Data collection

Data were extracted from included articles and entered into specifically designed spreadsheets. The following data fields were extracted: full citation, year, country and context of the research, study design, sample size, participant inclusion/exclusion criteria, intervention/s, controls, time period of intervention, outcome measures used, outcome time points, and outcome results. For six studies data had not been published in a form that enabled meta-analysis and these authors were contacted to request further data. This resulted in the inclusion of unpublished data from two author groups [21, 22].

### Risk of bias for individual studies

Two reviewers (AW, ET or JL) independently assessed the risk of bias using the Cochrane Risk of Bias tool and reached consensus decisions. Assessment scored papers as high, low or unclear risk of bias in the following domains: randomisation sequence generation, concealment of allocation, blinding of participants and personnel, blinding of outcome assessment, incomplete outcome data, and selective outcome reporting. Risk of bias results are reported in Table 2 for information only as they did not influence exclusion from the review.

### Summary measures

We reported the review’s summary measures for residential aged care admission and mortality as risk difference and 95% confidence intervals (CI), and quality of life as standardised mean differences with random effects and 95% CI.

### Data synthesis

Given the broad nature of this review, a high degree of heterogeneity between studies was anticipated and found, making narrative synthesis of the data the most appropriate method/approach for many studies. A meta-analysis was conducted where sufficient homogeneity existed between two or more studies, taking into consideration comparators, outcomes and assessment time-points. The synthesis also presents the findings for intervention sub-groups according to broad intervention types or target groups where possible.

For analysis of dichotomous outcomes, risk differences were calculated using the number of events within the overall participant number for each group. Risk differences give an absolute effect that is more readily interpreted to reflect the risk of an outcome across the two groups. For continuous outcomes we used standardized mean differences, calculated from the mean and standard deviation of each group outcome, to allow for some heterogeneity in the specific measures.

For both forms of outcomes we calculated 95% CI and provided levels of significance to allow for interpretation, as well as an evaluation of the statistical heterogeneity across the pooled studies using the I^2^ statistic. All calculations were performed in Revman 5.3 [23].

## Results

A flow diagram (see Fig. 1) reports the selection process and reasons for exclusion.

### Study selection

The database searches found 55,221 citations, and 50 citations were gleaned from hand searched reference lists and other sources. From these, 32 articles (reporting 31 trials) published between 2000 and 2018 met the review’s selection criteria (see Fig. 1).

The characteristics and summarized outcomes for included RCTs are reported in Table 1.

**Table 1:** Characteristics and summarized outcomes

First author, date, country, study design	Inclusion criteria	No. of participants & mean age	Intervention	Duration of intervention & visit frequency	Outcomes & time period	Shortened results (intervention group c/f control)
Complex interventions						
Beland 2006 Canada RCT (2 articles)	Aged ≥64 years; community-dwelling; French or English; participating caregiver; functional disability; no pending RAC admission.	SIPA *n* = 606 Control *n* = 624 82 years	SIPA (System of Integrated Care for Older Persons): multidisciplinary teams with full clinical responsibility for delivering comprehensive community-based care & coordination across health and social welfare sectors	Average length of enrolment was 572 days over the 662 day trial	Institutional admission, ED utilized, Skilled RAC utilized. Hospital utilized, Number in hospital waiting RAC placement Baseline & 12 months.	Skilled RAC admissions →. Decreased waiting in hospital for RAC place. Utilization or costs for ED, or acute hospital →.
Dalby 2000 Canada RCT	Functional impairment or hospital admission or bereavement in the previous 6 mths; aged ≥70 yrs.; at risk of sudden deterioration in health; community dwelling; not involved in other studies or previous nurse visits.	Intervention *n* = 73 (79.1 yrs) Control *n* = 69 (78.1 yrs)	Nurse led assessment, care plan development and case management	14 months Varied as needed by individual	RAC admission; Health services utilization; ED visits; Hospital admissions Baseline, 14mths f/up.	RAC admission →. Health services utilization, visits to ED or overnight hospital admissions →.
Eloniemi-Sulkava 2001} Finland RCT	Aged ≥65 yrs.; dementia; living at home with informal caregiver; no other severe diseases that might lead to institutionalization.	Intervention *n* = 53 (78.8 yrs) Control *n* = 47 (80.1 yrs)	Comprehensive, case managed dementia support for client and carer (nurse led)	2 years Individualised frequency of contacts from once a month to 5 times a day.	RAC admission; Deaths Baseline, 1 & 2 yr. f/up.	RAC admission at 12 months was reduced. RAC admission at 2 yrs. → . Deaths →.
Eloniemi-Sulkava 2009 Finland RCT	Spouse caring for a partner with dementia at home; dementia diagnosis; no other severe disease with prognosis < 6 months.	Intervention *n* = 63 (78 yrs) Control n = 62 (77 yrs)	Comprehensive, case managed dementia support for client and carer (nurse & geriatrician led)	Maximum2 yrs– varied phased recruitment Individualised, could be frequent contact	Admitted to RAC Deaths Baseline, 6, 12 & 24 mths f/up.	RAC admission at 12mths → . Reduced RAC admission at 18mths. RAC admission at 2 yrs. → . Deaths at 24 months →.
Hammar 2007 Finland Cluster RCT	Aged ≥65 years; discharged from hospital back home with home care services; primary admission diagnosis was not cancer, dementia or psychiatric; able to answer mental status-test	Intervention *n* = 354 Control *n* = 314 81.7 yrs	Generic community care and case management (IHCaD-practice) commencing with hospital discharge planning, and tailored to municipalities needs.	6 month program Frequency unclear	Admitted to RAC; Deaths; Finnish version of ADL; hospital care; HRQoL (NHP & EQ-5D) Baseline, 3 wks & 6 mths f/up	RAC admission at 6mth → . Deaths at 6mths → . ADL change at 6mths → . The EQ-5D change at 6mths → . Hospital care at 3wks & 6mth → .
Mahoney 2007 USA RCT	Aged ≥65; independently living; history of 2 falls in past year, or 1 injurious fall in past 2 yrs., or gait & balance problems; caregiver in the home.	Intervention *n* = 174 (79.6 yrs) Control *n* = 175 (80 yrs)	Multi-factorial falls prevention intervention linking participants to existing medical care & service networks.	12 month program 2 visits in-home first 3 weeks, then monthly phone contact	RAC admissions; RAC days; Mortality; Barthel scores; Depression (GDS); Hospitalisation; Hospital days; Baseline, 12 mth f/up.	RAC admissions →. Fewer RAC days per year. Hospitalisation →. Hospital days →. Barthel scores →. Mean change in GDS score →.
Markle-Reid 2013 Canada RCT	Trial 1: > 75 years; eligible for personal support services; not eligible for nursing. Trial 2: > 75 years; eligible for personal support services; at risk for falls. Trial 3: confirmed diagnosis of stroke or transient ischaemic attack in past 18 months; eligible for home care services	Trial 1: Intervention n = 144 Control *n* = 144 83.8 yrs. Trial 2: Intervention *n* = 54 Control *n* = 55 84 yrs. Trial 3: Intervention *n* = 52 Control *n* = 49 74.3 yrs	3 different health promotion, disease prevention interventions targeting functional decline and frailty	Trial 1: 5 home visits over 6mths (nursing) Trial 2: median of 19.5 home visits by the interD team over 6mths Trial 3: median of 24 home visits by the interD team over 12mths	Long term care (RAC) admissions; Mortality; SF-36 score; Depression (CES-D). Baseline, 6 & 12 mths f/up.	Trial 1: RAC long-term care →. Mortality →. Improved SF-36 mental health & emotional components. Reduced depression. Number of falls →. Trial 2: RAC long-term care →. Mortality →. SF-36 scores →. Reduced falls Trial 3: RAC long-term care →. Mortality →. SF-36 scores →. Number of falls →.
Nakanishi 2018 Japan Cluster-RCT	Aged > 65 years; Home-living patients with diagnosed dementia	Intervention *n* = 141 (83.7 yrs) Control *n* = 142 (84.9 yrs)	Challenging behaviour dementia training for care professionals; assessment of client behaviours & unmet needs; action plan; individualised multi-D treatment; behaviour monitoring; case management	6mth program Could have frequent contacts	RAC placement; Mortality; Challenging behaviour (NPI-NH); Pain (Abbey pain scale); Cognition (SMQ); Barthel Index for ADLs; Medication use Baseline, 6mths	RAC admission →. Mortality →. Challenging behaviours significantly improved in intervention group. Other outcomes →.
Phung 2013 Denmark RCT	Home-living patients diagnosed within the past 12 months with AD, mixed AD with vascular component or Lewy body dementia; ≥50 years; MMSE score ≥ 20; having one participating primary caregiver. All patients met DSM-IV criteria for dementia, NINCDS-ADRDA criteria for probable AD or McKeith criteria for Lewy body dementia. No severe somatic or psychiatric comorbidities	Intervention *n* = 163 (76.5 yrs) Control *n* = 167 (75.9 yrs)	Counselling, training, information and support for patients with mild dementia and their caregivers (DAISY)	8-12mths program Phone contact every 3–4 weeks, 7 individual sessions, 5 group sessions	Patients: RAC admissions; MMSE; Cornell Depression Scale (CDS); Health related QoL (EQ-VAS); QoL-AD; EuroQoL EQ-5D; Neuropsychiatric Inventory (NPIQ); ADSC-ADLs; Mortality; Carers: Geriatric Depression Scale (GPS); Health related QoL (EQ-VAS); Baseline, 6, 12 & 36 mths f/up.	RAC admission →. MMSE changes →. CDS changes →. EQ-VAS changes→. QoL-AD changes→. NPIQ changes →. ADSC-ADL changes →. Mortality →.
Samus 2014 USA RCT	Aged 70+ yrs.; English-speaking; community-residing; reliable partner; dementia or other cognitive disease; > 1 unmet need on JHDCNA; not in crisis (no signs of abuse, neglect, risk of danger to self/others)	Intervention *n* = 110 (84.0 yrs) Control *n* = 193 (83.9 yrs)	Interdisciplinary team case management, care planning, education & support for people with dementia (MIND)	18mth program Monthly contact	Days at home; RAC placement; Mortality; QOL-AD; ADRQL-40; QOL-AD-Informant; Neuropsychiatric Inventory (NPIQ); Depression (CSDD) Baseline, 9 & 18 mths f/up.	Increase in mean days at home. Reduced RAC placement or death. [RAC admit not reported separately] Improved self-reported QOL (QOL-AD). Proxy rated QOL (ADRQL-40; QOL-AD-Informant) → . NPS (NPI-Q), or participant depression (CSDD) Intervention →.
Senior 2014 New Zealand RCT	Age ≥ 65 years (≥55 years for Māori); at high risk of institutionalisation but not placed; communicate in English.	Intervention n = 52 (81.9 yrs) Control *n* = 53 (83.6 yrs)	Case-managed restorative care service delivered in short-stay residential aged care facilities and at participants’ residences (Promoting Independence Programmes)	2 yr. program Could have frequent contacts	RAC placements; Deaths Baseline, 24 mths f/up.	RAC placements →. Deaths →.
Shapiro 2002 USA RCT	Elders on a waiting list for community aged care; scored moderate risk based on Axs of chronic health conditions, ADL limitations, & other measures of physical & psychological impairment.	Intervention *n* = 40 (77.7 yrs) Control *n* = 65 (77.1 yrs)	Case managed, early intervention social service program for low-income elders	18mth program Monthly contact	Institutionalised (RAC admission); Deaths; Depression (12-item Center for Epidemiological Studies Depression scale) Baseline, 3, 6, 9, 12, 15 & 18 mths f/up.	RAC admission →. Death →. Improved OR for RAC admission or death. Depression →.
Spoorenberg 2018 Netherlands RCT	Age > 75 years; registered with a participating GP; not receiving other integrative care	Intervention *n* = 747 (80.6 yrs) Control *n* = 709 (80.8 yrs) Stratified by risk profile	Individualised program to maintain health & independence. Case managed, care plans, self management, information sessions, targeted support (EMBRACE)	12 mths Could be frequent contacts	Institutionalised (RAC admission); Deaths; Health status (EQ-5D-3 L, INTERMED-E-SA, GFI, Katz-15); Wellbeing (GWI, QoL); Self management (SMAS = 30), PIH-OA) Baseline, 12mths	RAC admission →. Death →. Deterioration in ADLs in intervention group (p = 0.04) Other health status → Wellbeing →. Self management →.
Single focus interventions						
Byles 2004 Australia RCT	Veterans or war widows with full entitlements from DVA; aged ≥70 years; community dwelling.	Intervention *n* = 942 Control *n* = 627	Annual or 6 monthly in-home assessments, provision of health materials, and report and liaison with GP.	3 year program	Permanent admission to a RAC; deaths; SF-36 scores; Hospital admissions; Baseline, 1 yr., 2 yr. & 3 yr. f/up.	Increased permanent RAC admission. Number of deaths →. SF-36 scores →. Hospital admission →.
Gill 2002 USA RCT	Aged ≥75 years; community dwelling; physically frail; can walk; speak English; MMSE score ≥ 20; life expectancy of > 12 mths; no major health event <6mths	Intervention n = 94 (82.8 yrs) Control *n* = 94 (83.5 yrs)	Home exercise program led by physical therapist to improve mobility and balance	6 month program 16 visits over 6 months - varied	RAC admissions; Deaths; Baseline, 3, 7 & 12 months f/up	RAC admission by 12 mth f/up →. Number of days spent in a RAC by 12mths → . Deaths during 12 mth → .
Hebert 2001 Canada RCT	On Quebec Health Insurance Plan list; Aged > 75 years; community dwelling; born between 1 December & 30 April; spoke English or French	Intervention *n* = 250 (80.2 yrs) Control *n* = 253 (80.3 yrs)	Nursing assessment, report and recommendations to GP, monthly phone review	1 year program Monthly contact	Admitted to RAC; Health service utilization; Functional Measurement Autonomy (SMAF); General Wellbeing Schedule (GWBS); Social provisions scale (SPS); Deaths Baseline, 12 mths f/up.	Admission to RAC → . Mean scores in SMAF, GWBS, SPS → . Deaths Intervention →. Health service utilization →.
Holland 2005 UK RCT	Aged ≥80 yrs.; emergency hospital admission; discharged to own home or warden controlled accommodation; prescribed ≥2 drugs on discharge; no dialysis treatment.	Intervention *n* = 429 (85.4 yrs) Control *n* = 426 (85.5 yrs)	Home visit for medication review and education by pharmacist following hospital discharge	6–8 week program 2 visits	RAC admissions; Mortality; EQ-5D (QoL); Emergency readmissions; Baseline, 3 & 6 mths f/up.	RAC admissions →. Reduced emergency readmissions. Increased GPs home visits. Deaths →. Change in QoL EQ-5D scores →.
Lenaghan 2007 UK RCT	> 80 years; living in own home; prescribed ≥4 daily medicines; & ≥ 1 criteria present: living alone; mental confusion vision; hearing impairment;prescribed medicines associated with medication-related morbidity; or prescribed > 7 regular oral medicines.	Intervention *n* = 68 (84.5 yrs) Control *n* = 66 (84.1 yrs)	Home visits by pharmacist for medication review and education	2 visits in 8 weeks	RAC admissions; Deaths; EQ-5D (QoL) Unplanned hospital admissions Baseline, 6 mths f/up.	RAC admissions →. Deaths →. The EQ-5D scores →. Unplanned hospital admissions →.
Luukinen 2006 Finland RCT	Home dwelling; history of recurrent falls in past year, or at ≥1 risk factor for disability in ADLs or mobility.	Intervention n = 243 Control *n* = 243 88 yrs	Community exercise program to prevent disability	18–24 month program Bimonthly contact	RAC admission; Mobility score; Balance impairment; ADL Baseline, end of intervention & f/up.	RAC admission →. Severe mobility restrictions at f/up →. Reduction in impaired balance. Improved mobility scores. ADL score improvement →.
Newbury 2001 Australia RCT	≥75 years; attending 1 of 6 GP practice sites; community dwelling; no dementia diagnosis	Control n = 50 (80.76 yrs) Intervention *n* = 50 (78.96 yrs)	Two annual 75+ Health Assessments with report back to GP	2 year program Annual assessments	Institution (RAC) admissions; Barthel ADL; Self-rated health; Deaths; Folstein MMS; GDS 15; SF-36 Baseline, 12 mths f/up.	RAC admission →. Barthel ADL → . Self-rated health →. Deaths →. Folstein MMS Intervention →. GDS 15 Intervention →. SF-36 → .
Pardessus 2002 France RCT	Aged ≥65 yrs.; hospitalized for falling; discharged home; no cognitive impairment; fall not secondary to medical or therapeutic problems; access to phone.	Intervention n = 30 (83.51 yrs) Control *n* = 30 (82.9 yrs)	Single occupational therapy home visit to address risk of falls	12mth program 1 x home visit	RAC admissions; Functional autonomy measurement system (SMAF); Total ADL; Total IADL; Recurring fall; Hospitalization for fall; Hospitalization for another cause; Deaths Baseline, 6 & 12 mths f/up	RAC admission →. Total SMAF 6–12 months Intervention →. Total ADL scores at 6 or 12mths → . Total IADL scores at 6 or 12mths → . Total SMAF at 6 or 12mths → . Recurring fall →. Hospitalization for fall →. Hospitalisation for another cause →. Death →.
Spice 2009 United Kingdom Cluster RCT	Aged ≥65 yrs.; community living; ≥2 falls in previous year; not presenting to ED with most recent fall; life expectancy > 1 yr.; abbreviated mental test score ≥ 7; English speakers.	Controls *n* = 159 (83 yrs) Primary care *n* = 136 (83 yrs) Secondary care *n* = 210 (81 yrs)	Primary care intervention group – GP assessment to identify falls risk; referrals as needed. Secondary care intervention group - multi-disciplinary Day Hospital falls prevention assessment with referrals as needed	12 months Monthly contact	RAC admissions; Falls; Fall-related hospital admissions; Mobility (Get up & go test) Baseline, 12 mths f/up.	Admission to RAC → Reduced falls.in Secondary Care Gp. Falls in Primary Care Gp → . Mobility score →. Fall-related hospital admissions →.
Thomas 2007 Canada RCT	Aged ≥75 years; no formal home care services; receiving informal care; not in RAC or other long term care; has a primary caregiver; English speaking; mentally competent.	Intervention (1) n = 175 (80.7 yrs) Intervention (2) *n* = 170 (80.4 yrs) Control *n* = 175 (80.7 yrs)	Annual functional assessments with either (1) elders and carers only given results only, or (2) also offered help with referrals	4 year program Annual contact	Institutional (RAC) admissions; Deaths; Self-efficacy; Self-rated health status; Caregiver burden Baseline, yr1, yr2, yr3, yr4.	RAC admissions →. Deaths →. Self-efficacy →. Self-rated health status →. Caregiver burden →.
Vass 2005 Denmark RCT	Aged 75-80 yrs.; Non-institutionalised;	Intervention *n* = 1798 Control *n* = 1688 75 yr. and 80 yr. cohorts	Educational program for healthcare professionals and GPs in geriatric assessment and recognising early functional decline	3 year program 6 monthly contact	RAC admissions; Mortality; Functional ability Baseline, 3 yr. f/up.	RAC admissions →. Mortality →. Improved functional ability in the 80 yr. old. Improved functional ability in the 75 yr. olds.
OTHER RCTs (not clearly complex nor minimal interventions)						
Caplan 2004 Australia RCT	Aged ≥75 yrs.; discharged from ED; community dwelling.	Intervention *n* = 370 (82.1 yrs) Control *n* = 369 (82.4 yrs)	In-home assessment following ED presentation, with 28 days community support from hospital-based MultiD team	4 weeks	RAC admission; ED admission; Hospital admission; Mortality. Baseline, 3, 6, 12 & 18 months f/up.	RAC admission →. Reduced emergency admission to hospital. Increased time to first ED admission. Mortality →.
Kono 2012 Japan RCT	Aged ≥65 years; need support to live at home; living at home; not used formal long-term care services for the past 3 months.	Intervention *n* = 161 (80.3 yrs) Control *n* = 162 (79.6 yrs)	Routine preventive home visits 6 monthly	Every 6 months for 2 years	Institutional admissions (RAC or group home); Deaths; Admitted to hospital; Decline in ADLs; Depression Baseline, 1 & 2 yr. f/up	Institutionalized at 2 yr. → . Deaths at 2 yrs. → . Hospital admissions →. Less decline in ADLs ability. Reduced depression. Increased utilisation of community long-term care.
Kono 2004 Japan RCT	Aged > 65; living at home; walk independently; need some assistance to live in the community; went outdoors <3x/wk.	Intervention *n* = 59 (82.5 yrs) Control n = 60 (82.9 yrs)	Preventive home visits by public health nurses 3 monthly	Home visits every 3 months for 18 months	Living at home; RAC admissions; Mortality; ADLs; Social support; Functional status Baseline, 18 mths f/up.	Living at home →. Admitted to RAC → . Deaths →. Less declining ADLs. Social support →.
Rockwood 2000 Canada RCT	Frailty (concern about community living, or recent bereavement, or hospitalization, or acute illness); frequent physician contact; multiple medical problems; polypharmacy; adverse drug events; functional impairment or functional decline; diagnostic uncertainty.	Intervention *n* = 95 (81.4 yrs) Control *n* = 87 (82.2 yrs)	Implementation of Comprehensive Geriatrician Assessment recommendations by a mobile geriatric assessment team.	3mth program Range 1–6 contacts	RAC admissions; Goal Attainment Scale (GAS); Deaths Baseline, 3, 6 & 12 mths f/up.	RAC admissions →. Improved Goal Attainment (GAS). Deaths →.
Scott 2004 USA RCT	Aged ≥60 yrs.; ≥ 11 outpatient clinic visits in the prior 18 months; ≥1 chronic conditions; able to attend clinic; no serious cognitive impairment.	Intervention n = 14 (74.2 yrs) Control *n* = 149 (74.1 yrs)	Monthly group meetings for education, support & health review led by patients’ GP and a nurse	2 yr. program Monthly contact	Skilled nursing facility (RAC) admissions; Hospital admissions; Pharmacy services; Health facility visits; ADLs; Self-reported Quality of life; Baseline, 24 mths f/up.	RAC admissions →. Reduced hospital admissions. Reduced emergency visits. Improved self-reported quality of life. Increased self-efficacy. ADLs →. Pharmacy services →. Hospital outpatient visits →.
Sommers 2000 USA Cluster RCT	Aged > 65; not in RAC; 1+ visit to GP past 3mths; English speaking; Indep in mobility toileting feeding; Dependant in 1+ IADL; 2+ chronic conditions; not terminally ill; no dementia or metastatic disease.	Intervention *n* = 280 (77 yrs) Control *n* = 263 (78 yrs)	Collaborative care from a GP, nurse and social worker for chronically ill elders (chronic disease self-management model)	3 yr. program Contact at least 6 weekly	RAC admissions; Symptom scale; SF-36; Health Activities Questionnaire (HAQ); Depression (GDS); Medication count; Nutrition checklist; Hospital admissions; GP office visits; Deaths; Social activities; Baseline, yr1 & yr2	RAC admissions →. Reduced hospital admissions. Fewer GP office visits/yr. Symptom scale, SF-36, HAQ, GDS, Medication count and Nutrition checklist Intervention →. Deaths →.
Stuck 2000 Switzerland RCT	Community living; Age 75+; German speaking; not terminal disease	Intervention *n* = 264 82 yrs. Control *n* = 527 81.5 yrs	Annual geriatric assessments with quarterly preventative home visits by a nurse	3 yr. program 3 monthly contact	RAC admissions; Functional status; Mortality Baseline, 1 yr., 2 yr., 3 yr. f/up.	RAC admissions →. Dependent in ADL or iADLs →. Mortality →.
Van Hout 2010 The Netherlands RCT	Aged ≥75 yrs.; living at home; meet criteria for frailty	Intervention *n* = 331 (81.3 yrs) Control *n* = 320 (81.5 yrs)	Geriatric assessments by nurses, personalized care plans and preventative home visiting	18mth program 3 monthly contact	Institutional (RAC) admissions; Deaths; Hospital admissions; SF-36; ADL; IADL; Emergency visits Baseline, 6 & 18 mths f/up.	RAC admission →. Death →. Hospital admissions →. Emergency visits →. ADLs & iADLs →.

→ Indicates no significant difference between outcomes of intervention and control groups

AD-Alzheimer’s disease; ADL-activities of daily living; ADRQL-40-Alzheimer’s Disease Rated Quality of Life-40 item; ADSC-ADL-Alzheimer’s Disease Cooperative Study Activities of Daily Living Scales; CDS-Cornell depression scale; CES-D-Centre for Epidemiological Studies Depression Scale; CSDD-Cornell Scale for Depression in Dementia; DSM-IV- Diagnostic and Statistical Manual of Mental Disorders version 4; DVA-Department of Veterans Affairs; ED-emergency department; EuroQoL - A generic utility measure used to characterize current health states; EQ-5D-EuroQol five dimensions questionnaire; EQ-5D-3 L -EuroQoL-5D-3 level version; EQ-VAS-EuroQol Visual Analogue Scales; Folstein MMS-Folstein Mini-Mental State; GAS-goal attainment scale; GDS-geriatric depression scale; GFI −15 item measure of frailty; GP-General Practitioner; GWBS-general wellbeing schedule; GWI –Groningen Wellbeing Index; HAQ-health activities questionnaire; IADL-instrumental activities of daily living; IHCaD-practice-generic prototype of care/case management-practice; INTERMED-E-SA – measure of complexity of care needs; Katz-15 – meaure of ADL limitations; MMSE-mini-mental state examination; Multi-D-multidisciplinary; JHDCNA-Johns Hopkins Dementia Care Needs Assessment; RAC-Residential Aged Care; NINCDS-ADRDA- National Institute of Neurological and Communicative Disorders and Stroke and the Alzheimer’s Disease and Related Disorders Association; NPIQ -Neuropsychiatric inventory questionnaire; NPI-NH -Neuropsychiatric inventory questionnaire fro Nursing Homes; OR-odds ratio; PIH-OA –Partners in Health Scale; QoL-quality of life; QoL-AD -Quality of Life Scale for Alzheimer’s disease; RCT-randomised control trial; SF-36-36-item Medical Outcomes Study Short Form; SIPA-System of Integrated Care for Older Persons; SMAF-functional measurement autonomy system; SMQ –Short Memory Questionnaire; SMAS – Self-Management Ability Scale; SPS-social provisions scale

### Types of control conditions

Most RCTs described their control groups as receiving ‘usual care’. This was not explained in any detail and is likely to have differed across settings and across the 11 countries where the research was conducted (Canada, Finland, the United States (US), Japan, Denmark, New Zealand, the Netherlands, Australia, United Kingdom (UK), France, Switzerland). Four studies provided augmented usual care for control group participants such as additional educational and resource materials [24, 25], an education program [26], or an occupational therapy home assessment [27].

### Types of interventions and targeted participants

The majority of RCTs aimed to support older community-dwelling people who were at risk of functional decline and residential aged care admission. Some trials targeted people with specific conditions or risk factors such as dementia [24, 25, 28–30], recurrent falls [21, 27, 31–33], transition from hospital to community [32, 34–36], or polypharmacy [36–38].

Details of intervention elements were extracted from RCTs and entered into a table to assist our understanding of the studies’ interventions. This can be found in Additional file 2. While the types of interventions varied greatly and were frequently reported in very little detail, we were able to allocate many studies, but not all, to sub-groups. Most of these sub-groups are defined or described earlier under ‘Description of the intervention’. In addition we decided to consider the effect of complex interventions compared to minimal/simple interventions. Complex interventions (e.g. multifactorial preventative home visits) addressed multiple issues with case management, multi-disciplinary input and multiple participant contacts during the program. Complex intervention appeared to provide a higher intensity intervention (more elements of care and/or frequency of contact) than that provided in single focus intervention programs. Single focus interventions were provided by a single discipline, focused on one area of care and/or had very few participant contacts (e.g. an assessment with report to the general practitioner (GP), short term exercise program by a physiotherapist). The intervention sub-groups of studies were:

- **Complex interventions.** Thirteen RCTs (14 articles), with a total of *n* = 5,694 participants [21, 24, 25, 27–30, 34, 39–44].
- **Single focus interventions.** Eleven RCTs, with a total of *n* = 8,926 participants [22, 26, 31–33, 36, 37, 45–48].
- **Re-enablement or restorative care.** Seven RCTs (8 articles), *n* = 2,842 participants trialled interventions targeting people who had falls [21, 27, 32, 33]), broader mobility issues [26], or aimed for more general functional restoration [39, 40, 43]. Four of these RCTs (five articles) also appear in the complex intervention sub-group, and three in the minimal intervention sub-group.
- **Dementia specific interventions.** Four RCTs specifically targeted people with dementia and their family carers. Four of these studies fitted our criteria for complex interventions [24, 25, 28, 30]. One dementia study did not provide case management or refer participants to external support services, but did provide semi-individualised counselling, training and information to support people through the early months after dementia diagnosis [29].

No RCTs were found that tested ‘centre-based wellness programs’ or ‘consumer directed care’ as defined earlier under ‘Description of the intervention’.

All complex intervention studies identified case management as an element of the intervention they trialled, and so we did not analyse case management as a separate sub-group (case management usage within interventions can be found in Additional file 2).

Of the seven RCTs not allocated to a sub-group, one delivered a comprehensive chronic disease self-management intervention [49], one investigated support for the transition from hospital to community [35], one investigated GP led monthly group education and support meetings [50], and four others provided insufficient information about their intervention to enable sub-group allocation. [38, 51–53].

### Types of outcomes

All studies reported our primary outcome of admission to residential aged care during the study period. In addition two studies reported the outcome as days remaining at home [25], and days spent in residential aged care [27]. Some studies did not report the data in a form suitable for meta-analysis. In these cases we attempted to contact the original authors to request the data needed.

Secondary outcomes that were most commonly reported were considered in meta-analyses, these being mortality (reported in all RCTs), and various measures of quality of life reported in 12 studies [21, 25, 29, 34, 36, 37, 44–47, 50, 54].

Other outcomes are reported as narrative synthesis and in Table 1, including health service usage, functional ability, depression, mobility and falls, self-efficacy, and goal attainment.

### Risk of bias within RCTs

Appraisal of research quality revealed mixed risk of bias across the RCTs (see Table 2). Unfortunately the reporting in many articles was inadequate to determine whether or not risk of bias criteria had been met. Given the nature of the interventions, very few RCTs (9%) had been able to blind their participants and personnel to group allocation, however it was disappointing that blinding of outcome assessors was only reported in 51% of articles. Evidence of a low risk of bias from the randomization process was clear in only 29% studies; the concealment of group allocation was rarely reported. Accounting for all data had been of low risk in all but two studies, however only one study gave assurance that all outcomes had been reported as per their original protocol.

**Table 2:** Risk of bias

Author, year	Sequence generation	Allocation concealment	Blinding of participants and personnel	Blinding of outcome assessors	Incomplete outcome data	Selective outcome reporting
Beland 2006	L	?	?	L	L	?
Byles 2004	L	?	H	L	L	?
Caplan 2004	L	H	?	H	L	?
Dalby 2000	L	L	L	L	L	?
Eloniemi-Sulkava 2001	L	L	?	L	L	?
Eloniemi-Sulkava 2009	L	L	?	?	L	?
Gill 2002	L	?	?	L	L	?
Hammar 2007	L	?	?	H	L	?
Hebert 2001	L	?	L	L	L	?
Holland 2005	L	L	H	?	L	?
Kono 2004	L	?	L	L	L	?
Kono 2012	L	?	?	?	L	?
Lenaghan 2007	?	?	?	?	L	?
Luukinen 2007	L	?	?	L	L	?
Mahoney 2007	L	L	?	L	L	?
Markle-Reid 2013	L	?	?	?	?	?
Nakanishi 2018	L	L	H	H	L	?
Newbury 2001	L	L	H	?	L	L
Pardessus 2002	L	?	?	?	L	?
Phung 2013	?	?	?	L	L	?
Rockwood 2000	?	?	H	L	?	?
Samus 2014	L	?	H	L	L	?
Scott 2004	L	?	?	?	L	?
Senior 2014	?	L	H	L	L	?
Shapiro 2002	L	?	H	H	L	?
Sommers 2000	L	?	H	?	H	?
Spice 2009	L	?	?	?	L	?
Stuck 2000	L	L	?	L	L	?
Spoorenberg 2018	L	?	H	L	L	?
Thomas 2007	L	?	H	H	L	L
van Hout 2010	L	?	H	L	L	?
Vass 2005	L	?	H	H	L	?

Legend: *H* High risk, *L* Low risk? Unclear risk

## Results from RCTs

**Fig. 2:**
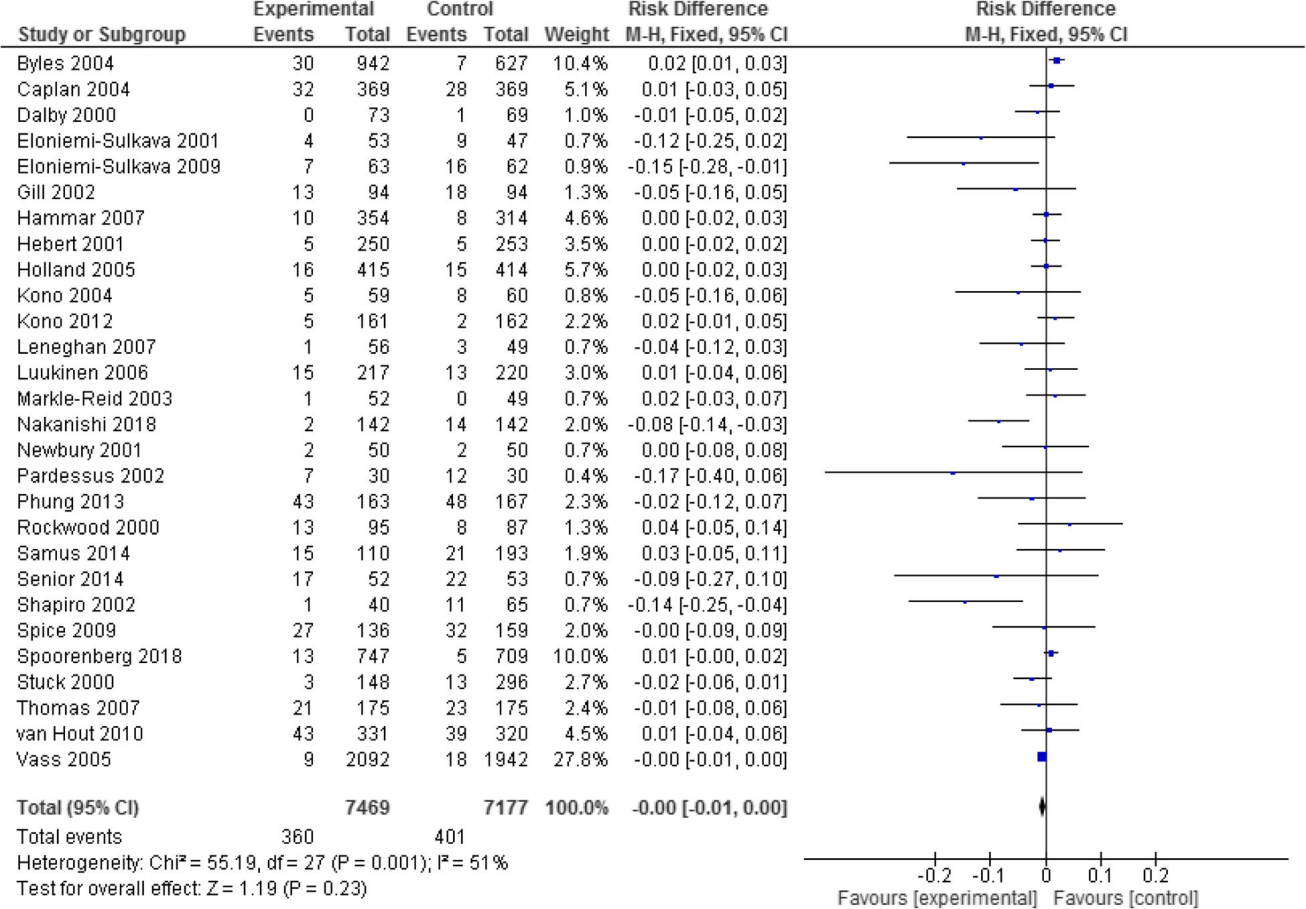
All interventions – outcome of residential aged care admissions

A summary of effectiveness for individual studies is provided in Table 1 and in the Forest Plots (Fig. 2 and Additional file 3).

### Meta-analysis results

#### Residential aged care admission outcomes

Twenty eight studies provided the relevant data to allow a meta-analysis for the primary outcome of residential aged care admission rates. An initial analysis of risk difference for all interventions together (compared to the control ‘usual care’) revealed no difference in risk of admission between the two groups (total admissions 360 from 7,469 participants in the intervention group, and 401 from 7,177 in the control group; RD -0.00 (95% CI -0.01, 0.0, *p* = 0.23; moderate heterogeneity I^2^ = 51%). See Fig. 2 for the complete forest plot.

#### Intervention sub-group analysis of residential aged care admission

Meta-analyses were also run for four sub-groups of (1) complex interventions (11 studies had appropriate data), (2) single focus interventions (11 studies), (3) dementia specific interventions (5 studies), and (4) restorative programs (5 studies).

**Fig. 3:**
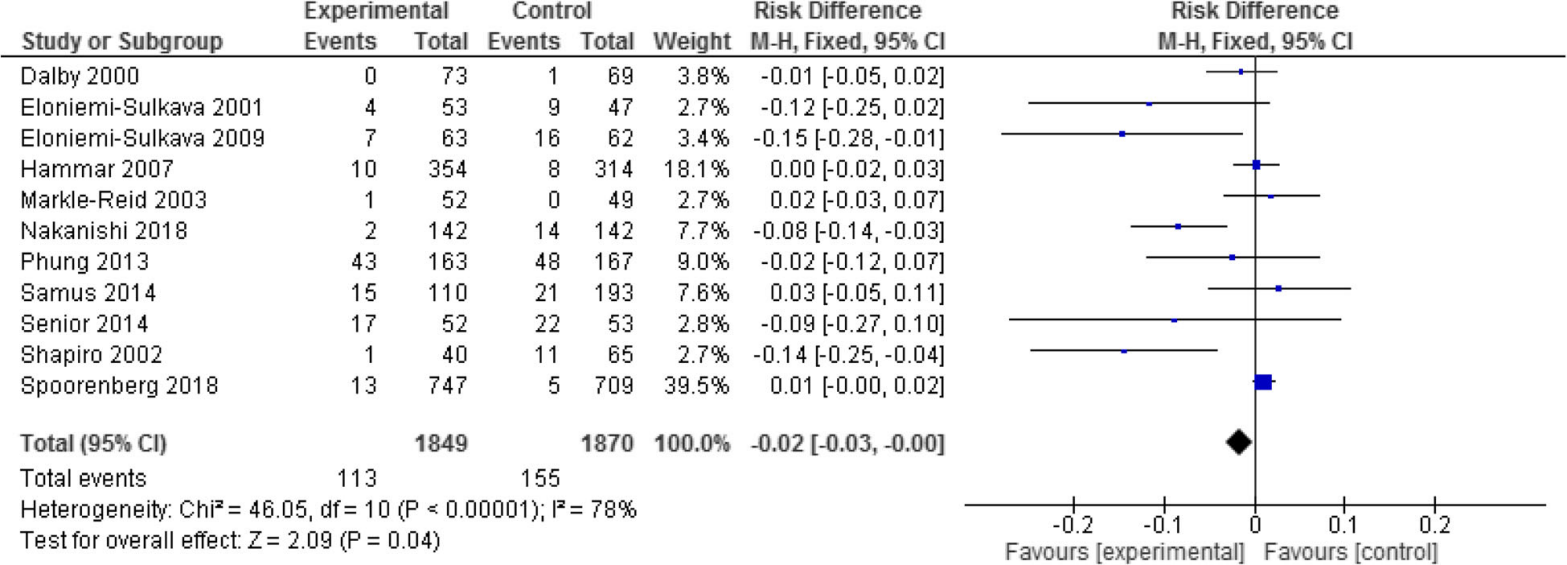
Complex interventions - outcome of residential aged care admissions

Considering the difference in risk of residential aged care admission in the intervention sub-groups: firstly for *complex interventions* the risk difference was significantly lower for the intervention participants by the order of 2% (RD -0.02; 95% CI -0.03, − 0.00; *p* = 0.04; I^2^ = 78%). This is illustrated in the Fig. 3 forest plot.

**Fig. 4:**
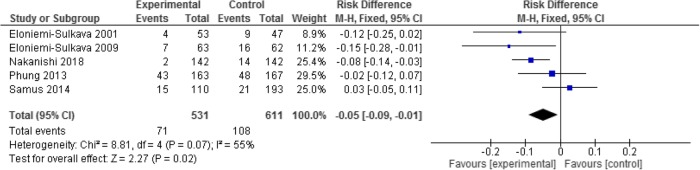
Dementia specific interventions – outcome of residential aged care admission

In the sub-group of *dementia-specific programs*, pooling the five studies showed a significant 5% risk reduction for residential aged care admission in the intervention group (71/531 participants), compared to the controls (108/611 participants) (RD -0.05; 95% CI -0.09, -0.01; *p* = 0.02; I^2^ = 55%) (See the forest plot in Fig. 4).

In contrast, the *single focus intervention* participants in 11 studies had no difference in risk of admission compared to the control (RD 0.00; 95% CI -0.01, 0.01; *p* = 0.71; I^2^ = 38%).

*Restorative programs* for people transitioning from hospitalisation to home, or at risk of falls (five studies) showed less residential aged care admission (53 from 445 participants) compared to control (65 from 446), however this was not significant (RD -0.03, 95% CI -0.07, 0.02; *p* = 0.23; I^2^ = 45%) (see forest plot in Additional file 3).

#### Mortality outcomes

Considering the secondary outcome of mortality, pooling 32 studies showed overall there was no difference in risk of dying between the intervention groups (deaths for all interventions combined 957/8,731, compared to controls 947/8,435) (RD -0.00; 95% CI -0.01, 0.01; *p* = 0.45; I^2^ = 1%) (see forest plot in Additional file 3). Sub-group analyses revealed no difference in mortality rates between any of the grouped interventions compared to the controls.

#### Quality of life outcomes

Quality of life was measured across a sufficient number of studies (*n* = 7) to warrant meta-analysis. Firstly considering all interventions compared to control, there was a standardized mean effect of 0.51, however this just failed to reach significance (95% CI -0.06, 1.09; *p* = 0.08) (see forest plot in Additional file 3). This included seven studies, with a total of 3,219 participants, but an I^2^ = 98% that is unacceptably high. We used random effects in response to this and also conducted a sensitivity analysis which revealed this was accounted for by one study reporting a stronger than usual effect in favour of the intervention group [25]. This was also the only intervention in the complex intervention sub-group analysis and not surprisingly showed a standardized mean difference of 3.38 (95% CI 3.02, 3.74; *p* < 0.000001).

In the sub-group of *dementia-specific programs,* quality of life outcomes from only one study were able to be analysed [25]. In this study the intervention group reported significantly improved quality of life compared to the control group (SMD 3.38, 95% CI 3.02, 3.74; *p* < 0.00001). (See forest plots in Additional file 3).

#### Duration of program

The duration of programs varied considerably. Interventions for studies within the complex intervention sub-group ranged from 6 months to 2 years duration (6 from 13 studies were > 18 months). The complex intervention RCTs with the strongest results for reducing residential aged care admission had data endpoints of 18 months to 2 years [24, 28, 41, 43]. Minimal intervention programs ranged from 8 weeks (medication reviews) to 4 years duration (the intervention being an annual assessment). More detail is available in Table 1 and Additional file 2: Table S1.

### Narrative synthesis of other RCT outcomes

Here we describe narratively outcomes that could not be examined by meta-analysis. The results are equivocal for these outcomes and further evidence is required before conclusions can be drawn on effectiveness.

#### Health service usage outcomes

In 10 RCTs from all intervention sub-groups (in 11 articles), there was no significant difference in health service usage, such as hospital admissions or emergency department attendance, between intervention groups compared to the controls [27, 31, 32, 34, 37, 39, 40, 42, 45, 46, 53, 54]. In contrast four trials with differing interventions reported significant reductions or shifts in health service usage: three in favour of intervention in reducing usage and one in favour of the control group.

- Caplan et.al. [35] reported 44.4% of their intervention group versus 54.3% of the control group had an emergency hospital admission over 18 months [Difference % (95% CI)] -9.9 (− 17.1 to − 2.7) *p* = 0.007.
- Scott et.al. [50] reported utilization as mean (standard deviation) hospital admissions per patient over 24 months, with significantly less utilization in the intervention group 0.44 ± 0.89 compared to controls 0.82 ± 1.7 (*p* = 0.013).
- Sommers et al. [49] reported over a 1 year period hospital readmissions for participants in the intervention group decreased from 6 to 4%, while the rate increased in the control group from 4 to 9% (*p* = 0.03)
- Holland et al. [36] reported a shift in health service utilization. At 6 months 234 hospital readmissions had occurred in the intervention group versus 178 in the control group (rate ratio = 1.30, (95% CI 1.07 to 1.58), *p* = 0.009. Concurrently GPs carried out 204 home visits in the intervention group and 125 in the control group, a difference of 43% (rate ratio = 1.43 (95% CI 1.14 to 1.80), *p* = 0.002).

#### Functional ability outcomes

Of the 13 studies reporting functional outcomes such as activity of daily living (ADL) measures, ten were unable to show significant difference between intervention and control group outcomes [27, 29, 32–34, 46, 47, 50, 51, 54]. Three studies, with differing interventions, did report significantly better functional outcomes in the intervention group compared to controls:

- In their small, initial study which was not clearly a complex nor a minimal intervention, Kono and colleagues [52] showed that intervention group subjects were less likely to show a decline in ADLs than control group subjects (*p* = .033).
- In their later RCT Kono’s group reported that for participants who had some dependency at baseline, those in the intervention group were significantly less likely to deteriorate over 2 years in their functional ADLs (*p* = .0311) or IADLs (*p* = .0114), compared to controls [53].
- Vass and colleagues [22] reported in their minimal intervention study that 85 year olds in their intervention group had better functional ability after 3 years than those in the control group [Odds Ratio 1.53 (95% CI 1.12–2.09), *p* = 0.008]; however there was no significant effect in younger participants.

#### Depression outcomes

Seven studies with differing types of interventions, considered measures of depression. Five of these reported no significant differences between intervention and control groups at follow-up [25, 27, 41, 47, 49]. Positive effects on depression were reported by two studies:

- Kono and colleagues [53] reported that for participants who had some ADL dependency at baseline, those in the intervention group (which was not clearly a complex nor a minimal intervention) were significantly less likely to deteriorate over 2 years in relation to experiencing depression (*p* = 0.0001)
- In one complex intervention arm conducted by Markle-Reid et.al. [21], intervention group participants had a statistically significant reduction in the Center for Epidemiologic Studies Depression Scale score than controls (− 2.72 (95% CI − 0.39 to − 5.07)), *p* = 0.022.

#### Mobility, balance, falls outcomes

No significant between-group differences were reported for the number of falls over 12 months in two RCTs [21, 32]. Spice and colleagues [31] reported no between-group mobility score differences. In contrast the 2006 study by Luukinen et.al. of a community exercise program (minimal intervention) reported positive change in mobility performance for the intervention subjects compared with the control (*p* = 0.013) and impaired balance affected fewer intervention subjects (45%) than controls (59%) (*p* = 0.015). Elements of the interventions differed across these studies [33].

#### Self-efficacy outcomes

Self-efficacy was measured in two trials. Thomas and colleagues [48], reported no significant group differences in self-efficacy outcomes in their minimal intervention trial, while Scott et.al. [50] reported a better self-efficacy rating only for ‘communication with their physician’ for the intervention group compared to controls (*p* = 0.03).

#### Goal attainment outcomes

The Goal Attainment Scale was used by Rockwood et.al. [38]. At 3 months follow-up the intervention group was more likely to have attained their goals, than the control group (*p* < 0.001).

## Discussion

In this paper we have systematically reviewed the published evidence of interventions to avoid or delay residential aged care admissions for older people living in the community, thus achieving our study objectives. This is the first known review to provide information on the elements of interventions and programs tested in published RCTs, and thus guide policy makers and healthcare providers on implementation of the more effective interventions.

It is clear that to reduce the risk of residential aged care admission requires multifactorial complex interventions as there is no evidence of significant effect from more minimal, single focus interventions. Furthermore, our meta-analysis has shown that complex interventions can reduce the risk of residential aged care admission for people with dementia. Given the complexity of aging with chronic health conditions including dementia, it is perhaps not surprising that interventions need to be multifaceted and complex in order to be effective. Within the context of an aging population it becomes even more important to understand which complex interventions are successful and which facets are necessary for success.

The most common elements in the complex intervention studies were the use of a comprehensive assessment process with good communication and liaison with GPs, individualised care plans and interventions with frequent client contact if required and regular reviews. Careful case management that included referrals to services not provided within the study intervention was also a common feature. In addition, developing skills and capacities within clients and/ or carers through education and training was a part of many complex intervention studies. What is less clear is who is best placed to deliver the assessment and case management, or whether there needs to be a multidisciplinary approach to service delivery. The complex intervention RCTs with the strongest results for reducing residential aged care admission had longer data endpoints (18 months to 2 years). It is likely that complex interventions need to be delivered over long time-frames to be influential and that follow-up at 18 months or longer is needed to capture effectiveness outcomes. The effectiveness of complex intervention may not attenuate over time.

While single focus interventions did not show a significant effect in reducing residential aged care admission, many of them showed a trend towards reducing admission and could be considered as elements within a multifactorial intervention project in future research. Examples were in-home medication reviews by a pharmacist [36], home safety assessments by occupational therapists [32], and mobility exercise programs by a physiotherapist [26]. A comprehensive complex intervention is likely to include similar specific interventions to the examples given.

Only five of 13 complex intervention studies specified that clients/carers were involved in decision making. The reporting quality of some studies may have failed to document a shared decision making process that had actually occurred. It is surprising that more emphasis has not been given to involvement of clients and/or carers in decision making given the emphasis in many policies and the preference for involvement demonstrated in other literature. It is likely that shared decision making will be required by future generations of older people as more informed consumers with higher expectations of services come to require service provision. Shared decision making fits with consumer driven models and optimises autonomy for clients and their support network [55]. Future studies could compare the outcomes for those specifically involved in shared decision making and planning with those who are not.

Consistent with earlier systematic reviews [56], our meta-analysis found no significant effect on mortality rates from any type of intervention. It may be that follow-up timeframes were too short to fully establish the impact of complex community interventions on extending survival days.

The only complex intervention studies that produced significantly better quality of life outcomes was one that focused on participants with dementia. Quality of life for people living in the community with dementia is known to be a complex and often distressing issue, and difficult to address [57]. Earlier studies have reported associations in this group between poor quality of life and unmet needs [58], including an inability to perform activities of daily living [59]. Individualised complex interventions similar to those in our included studies would appear to be a best-practice option for people with dementia.

### Limitations

As usual this systematic review may be affected by unknown publication bias. It is certainly limited by the methodological deficiencies in most of the included studies. Only one RCT demonstrated an overall low risk of bias and the remainder either had a high risk of bias or provided insufficient detail to determine bias. We note that considerable heterogeneity exists in several of the meta-analyses which suggests caution is needed when interpreting some results. There was insufficient detail in several included studies to fully understand the details of the intervention and/or the control conditions at a level that could be replicated. Future studies require careful planning and attention to risk and detailed reporting in order to strengthen the evidence base.

We did not search studies published in non-English journals or grey literature which may have caused us to miss relevant studies. We did not attempt a cost-effectiveness analysis – indeed there appears to be little evidence in the extant literature.

## Conclusions

Available evidence showed that multifactorial complex interventions in community aged care can significantly improve older adults’ ability to remain living at home and avoid residential aged care admission. While minimal or single focus interventions did not have a significant effect on delaying residential aged care admission, the direction of risk reduction in many of these studies favoured the intervention group.

There was no evidence of a significant effect on mortality rates or quality of life from any type of intervention compared to the controls, except a single study finding in favour of intervention for people with dementia.

Future studies are needed to investigate the specific components and costs of multifactorial community aged care interventions that are crucial in avoiding or delaying residential aged care admission for older adults, and that meet the preferences of older adults.

## Additional files

Additional file 1: Medline search string. (DOCX 12 kb)

Additional file 2: **Table S1.** Intervention elements. (DOCX 24 kb)

Additional file 3: Additional forest plots. (DOCX 266 kb)

## Data Availability

All data extracted or analysed during this study are included in this published article and its supplementary information files.

## Abbreviations

ADL: Activity of Daily Living
CI: Confidence Interval
GP: General Practitioner
PROSPERO: Prospective Register of Systematic Reviews
QoL: Quality of Life
RCT: Randomised Control Trial
