# Supplementary material for: The evidence for services to avoid or delay residential aged care admission: a systematic review
Source: BMC Geriatr. 2019 Aug 8;19:217. doi: 10.1186/s12877-019-1210-3 (PMC6686247; doi:10.1186/s12877-019-1210-3)
Supplement: Supplementary file 1 — Medline search string. (DOCX 12 kb) [file 12877_2019_1210_MOESM1_ESM.docx]

# **Additional file 1 Medline search string**

1. (aging or aged or ageing or elder* or senior or geriatric or old* adult or old* age).ti,ab.

2. ((community or independent) adj3 (dwelling or living or setting)).ti,ab.

3. "ag$ing in place".ti,ab.

4. home.ti,ab.

5. 2 or 3 or 4

6. (program* or model* or support* or activit* or service or intervention*).ti,ab.

7. preventative care.ti,ab.

8. (exercise or educat* or environment* or health or social or behavioural or pyscho*).ti,ab.

9. community care.ti,ab.

10. home care.ti,ab.

11. 7 or 8 or 9 or 10

12. 6 and 11

13. 1 and 5 and 12

14. limit 13 to (english language and yr="2000 -Current")
