# Supplementary material for: The evidence for services to avoid or delay residential aged care admission: a systematic review
Source: BMC Geriatr. 2019 Aug 8;19:217. doi: 10.1186/s12877-019-1210-3 (PMC6686247; doi:10.1186/s12877-019-1210-3)
Supplement: Supplementary file 2 — Table S1. Intervention elements. (DOCX 24 kb) [file 12877_2019_1210_MOESM2_ESM.docx]

**Additional File 2 Table S1 Intervention elements described**

|  | **Intervention**  **Element** | **Beland 2006** | **Byles 2004** | **Caplan 2004** | **Dalby 2000** | **Eloneumi-Sulkava 01** | **Eloneumi-Sulkava 09** | **Gill 2002** | **Hammar 2007** | **Hebert 2001** | **Holland 2005** | **Kono 2004** | **Kono 2012** | **Leneghan 2007** | **Luukinen 2006** | **Mahoney 2007** | **Markle-Reid2013** | **Nakanishi 2018** | **Newbury 2001** | **Pardessus 2002** | **Phung 2013** | **Rockwood 2000** | **Samus 2014** | **Scott 2004** | **Senior 2014** | **Shapiro 2002** | **Spice 2009** | **Spoorenberg 2018** | **Sommers 2000** | **Stuck 2000** | **Thomas 2007** | **Vass 2005** | **Van Hout 2010** |
| --- | --- | --- | --- | --- | --- | --- | --- | --- | --- | --- | --- | --- | --- | --- | --- | --- | --- | --- | --- | --- | --- | --- | --- | --- | --- | --- | --- | --- | --- | --- | --- | --- | --- |
| **Personnel** | General practitioners | X |  |  |  |  |  |  |  |  |  |  |  |  |  |  |  | X | X |  |  |  |  | X |  |  | X | X | X |  |  | X |  |
|  | Geriatricians, Physicians | X |  | X |  |  | X |  | X |  |  |  |  |  |  | X |  |  |  | X | X | X | X | X |  |  |  | X |  | X |  |  |  |
|  | Nurses | X | X | X | X | X | X |  | X | X |  | X | X |  | X | X | X | X | X |  | X | X | X |  | X | X | X | X | X | X | X |  | X |
|  | Occupational therapists | X | X | X |  |  |  |  |  |  |  |  |  |  | X |  | X |  |  | X |  | X |  |  | X |  |  |  |  |  |  |  |  |
|  | Physiotherapists | X | X | X |  |  |  | X |  |  |  |  |  |  | X | X | X | X |  |  |  | X |  |  | X |  |  |  |  |  |  |  |  |
|  | Dietitians | X |  |  |  |  |  |  |  |  |  |  |  |  |  |  | X |  |  |  |  | X |  |  |  |  |  |  |  |  |  |  |  |
|  | Social workers | X | X |  |  |  |  |  |  |  |  |  | X |  |  |  | X |  |  |  |  | X |  |  |  |  |  | X | X |  |  |  |  |
|  | Pharmacists | X |  |  |  |  |  |  |  |  | X |  |  | X |  |  |  |  |  |  |  |  |  |  |  |  |  |  |  |  |  |  |  |
|  | Psychologists |  | X |  |  |  |  |  |  |  |  |  |  |  |  |  |  |  |  |  |  |  |  |  |  |  |  |  |  |  |  |  |  |
|  | Speech pathologist |  |  |  |  |  |  |  |  |  |  |  |  |  |  |  | X |  |  |  |  | X |  |  |  |  |  |  |  |  |  |  |  |
|  | Audiologist |  |  |  |  |  |  |  |  |  |  |  |  |  |  |  | X |  |  |  |  | X |  |  |  |  |  |  |  |  |  |  |  |
|  | MultiD teams | X |  |  |  |  |  |  |  |  |  |  |  |  |  |  |  |  |  |  |  |  |  |  |  |  | X | X |  |  |  |  |  |
|  | Other support staff (aides, paid carers) | X |  |  |  |  |  |  | X |  |  |  |  |  |  |  | X | X |  |  |  |  |  |  | X |  |  |  |  |  |  |  |  |
| **Assessments** | Initial assessment | X | X | X | X | X | X | X | X | X | X | X | X | X | X | X | X | X | X | X | X | X | X | X | X | X | X | X | X | X | X |  | X |
|  | Review visits | X | X |  | X | X | X |  | X |  | X | X | X | X |  |  | X | X |  |  |  |  |  | X | X |  |  | X | X | X | X |  | X |
|  | Phone review |  | X |  | X | X |  | X |  | X |  |  |  |  | X | X |  |  |  | X | X |  | X |  | X |  | X |  | X |  |  |  |  |
|  | Home safety assessment |  | X |  | X |  |  | X |  |  |  | X |  |  |  | X |  |  |  | X |  |  |  |  | X |  | X |  | X |  |  |  |  |
| **Setting** | Client’s home | X | X | X | X | X | X | X | X | X | X | X | X | X | X | X | X | X |  | X | X | X | X |  | X | X | X | X | X | X | X |  | X |
|  | Hospital or ED | X |  | X |  |  | X |  | X |  |  |  |  |  |  |  |  |  |  | X |  |  |  |  |  |  |  |  |  |  |  |  |  |
|  | Health centre | X |  |  |  |  | X |  |  |  |  |  |  |  |  |  |  |  | X |  |  |  |  | X | X |  | X |  | X |  |  | X |  |
| **Intervention elements** | Case management | X |  |  | X | X | X |  | X |  |  |  |  |  |  |  | X | X |  |  |  |  | X |  | X | X |  | X |  |  |  |  | X |
|  | Referrals to other services | X |  | X | X |  | X |  | X | X |  |  |  |  |  | X | X | X |  |  |  |  | X |  | X |  | X | X |  |  | X |  | X |
|  | Care plan, recommendations | X |  | X | X | X | X |  | X |  |  | X | X |  |  |  | X | X |  |  |  |  | X |  | X | X |  | X | X | X |  |  | X |
|  | Liaison with GP | X | X | X | X | X | X |  |  | X | X |  |  | X |  | X |  |  | X |  |  | X |  |  | X |  |  | X |  |  |  |  |  |
|  | Medical interventions | X |  |  |  |  | X |  |  |  |  |  |  |  |  |  |  |  |  |  |  |  |  | X |  |  |  | X |  |  |  |  |  |
|  | Medication review |  |  |  |  |  |  |  |  |  | X |  |  | X |  |  |  |  |  |  |  |  |  |  |  |  |  |  |  |  |  |  |  |
|  | Nursing interventions |  |  | X | X |  |  |  |  |  |  | X |  |  |  |  |  |  |  |  |  |  |  | X |  |  |  |  |  |  |  |  |  |
|  | Physiotherapy & exercise interventions |  |  | X |  |  | X | X |  |  |  |  |  |  | X | X |  |  |  |  |  |  |  |  | X |  |  |  |  |  |  |  |  |
|  | Occupational therapy interventions |  |  | X |  |  |  |  |  |  |  |  |  |  | X |  |  |  |  |  |  |  |  |  | X |  |  |  |  |  |  |  |  |
|  | Individualized interventions | X |  | X |  | X | X |  |  |  |  |  |  |  |  |  |  | X |  |  |  | X |  |  |  |  |  | X |  |  |  |  |  |
|  | Hospital discharge planning |  |  | X |  |  |  |  | X |  |  |  |  |  |  |  |  |  |  |  |  |  |  |  | X |  |  |  |  |  |  |  |  |
|  | Personal care |  |  |  |  |  |  |  |  |  |  |  |  |  |  |  |  |  |  |  |  |  |  |  |  | X |  |  |  |  |  |  |  |
|  | Rehabilitation | X |  |  |  |  | X | X |  |  |  |  |  |  |  |  |  |  |  |  |  |  |  |  | X |  |  |  |  |  |  |  |  |
|  | Social or peer support | X |  |  |  |  | X |  |  |  |  |  |  |  |  |  |  |  |  |  | X |  |  | X |  |  |  |  |  |  |  |  |  |
|  | Dementia support |  |  |  |  | X | X |  |  |  |  |  |  |  |  |  |  | X |  |  |  |  | X |  |  |  |  |  |  |  |  |  |  |
|  | Carer support, respite |  |  |  |  | X | X |  |  |  |  | X |  |  |  |  |  |  |  |  | X |  |  |  |  | X |  |  |  |  |  |  |  |
|  | Counselling |  |  |  |  | X |  |  |  |  |  |  |  |  |  |  |  |  |  |  | X |  | X |  |  |  |  |  |  | X |  |  |  |
|  | Home help (homemaking) | X |  |  |  |  |  |  |  |  |  | X |  |  |  |  |  |  |  |  |  |  |  |  |  | X |  |  |  |  |  |  |  |
|  | Home delivered meals |  |  |  |  |  |  |  |  |  |  |  |  |  |  |  |  |  |  |  |  |  |  |  |  | X |  |  |  |  |  |  |  |
|  | Assistive/safety equip’t, medical supplies |  |  |  |  |  |  |  |  |  | X |  |  |  |  | X |  |  |  |  |  |  |  |  |  | X |  |  |  |  |  |  |  |
|  | Medical transport |  |  |  |  |  |  |  |  |  |  |  |  |  |  |  |  |  |  |  |  |  |  |  |  | X |  |  |  |  |  |  |  |
|  | Rapid response / on call | X |  |  |  | X |  |  |  |  |  |  |  |  |  |  |  |  |  |  |  |  |  |  |  |  |  |  |  |  |  |  |  |
|  | Education/training to patients/carers |  |  |  |  | X | X |  |  |  | X |  |  | X |  |  |  |  |  |  | X |  | X | X |  |  |  | X |  | X |  |  |  |
|  | Chronic disease self-management program |  |  |  |  |  |  |  |  |  |  |  |  |  |  |  |  |  |  |  |  |  |  |  |  |  |  | X | X |  |  |  |  |
|  | Information provision to patients/carers |  | X |  |  | X | X | X |  |  |  |  |  |  |  |  |  |  |  |  | X |  | X |  |  |  |  |  |  |  |  |  |  |
|  | Training for health professionals |  |  |  |  |  |  |  | X |  |  |  |  |  |  |  |  | X |  |  |  |  |  |  |  |  |  | x | X |  |  | X |  |
|  | Interventions unclear |  |  |  |  |  |  |  |  |  |  |  |  |  |  |  | X |  |  |  |  | X |  |  |  |  |  |  |  |  |  |  |  |
| **Decisions** | Client involved |  |  |  | X |  | X |  | X |  |  |  |  |  |  |  |  |  |  |  |  |  |  |  |  | X |  | X |  |  | X |  | X |
|  | Carer involved |  |  |  | X |  | X |  | X |  |  |  |  |  |  |  |  | X |  |  |  |  |  |  |  | X |  |  |  |  | X |  |  |
|  | Clinical team meetings |  |  | X |  |  |  |  |  |  |  |  |  |  |  |  |  | X |  |  |  | X |  |  |  |  |  | X | X | X |  |  |  |
| **Philosophy** | Integrated care across health sectors | X |  |  |  |  |  |  | X |  |  |  |  |  |  |  |  | X |  |  |  |  |  |  |  |  |  | X |  |  |  |  |  |
|  | Client-centered |  |  |  |  |  | X |  |  |  |  |  |  |  |  |  |  |  |  |  |  |  |  |  |  |  |  | X |  |  |  |  |  |
|  | Dementia specific |  |  |  |  | X | X |  |  |  |  |  |  |  |  |  |  | X |  |  | X |  | X |  |  |  |  |  |  |  |  |  |  |
|  | Applied EBP/ guideline | X |  |  |  |  |  |  |  |  |  |  |  |  |  |  | X |  |  |  |  |  |  |  |  |  |  |  |  |  |  |  | X |
| **Frequency of contact** | Annual or single |  | X |  |  |  |  |  |  |  |  |  |  |  |  |  |  |  | X | X |  |  |  |  |  |  |  |  |  |  | X |  |  |
|  | Monthly |  |  |  |  |  |  |  |  | X | X |  |  | X |  | X | X |  |  |  | X | X | X | X |  | X | X | X |  |  |  |  |  |
|  | 3 monthly |  | X |  |  |  |  |  |  |  |  | X |  |  |  |  |  |  |  |  |  |  |  |  |  |  |  |  |  | X |  |  | X |
|  | 6 monthly |  | X |  |  |  |  |  |  |  |  |  | X |  |  |  |  |  |  |  |  |  |  |  |  |  |  |  |  |  |  | X |  |
|  | Weekly - fortnightly |  |  | X |  |  |  | X |  |  |  |  |  |  | X |  |  |  |  |  |  |  |  |  |  |  |  |  |  |  |  |  |  |
|  | 6 weekly |  |  |  |  |  |  |  |  |  |  |  |  |  |  |  |  |  |  |  |  |  |  |  |  |  |  |  | X |  |  |  |  |
|  | Individual, could be very frequent | X |  |  | X | X | X |  |  |  |  |  |  |  |  |  |  | X |  |  |  |  |  |  | X |  |  | X |  |  |  |  |  |
|  | Unclear |  |  |  |  |  |  |  | X |  |  |  |  |  |  |  |  |  |  |  |  |  |  |  |  |  |  |  |  |  |  |  |  |
| **Length of Program** | 4 weeks |  |  | X |  |  |  |  |  |  |  |  |  |  |  |  |  |  |  |  |  |  |  |  |  |  |  |  |  |  |  |  |  |
|  | 8 weeks |  |  |  |  |  |  |  |  |  | X |  |  | X |  |  |  |  |  |  |  |  |  |  |  |  |  |  |  |  |  |  |  |
|  | 3 months |  |  |  |  |  |  |  |  |  |  |  |  |  |  |  |  |  |  |  |  | X |  |  |  |  |  |  |  |  |  |  |  |
|  | 6 months |  |  |  |  |  |  | X | X |  |  |  |  |  |  |  | X | X |  |  |  |  |  |  |  |  |  |  |  |  |  |  |  |
|  | 12 months |  |  |  |  |  |  |  |  | X |  |  |  |  |  | X | X |  |  | X | X |  |  |  |  |  | X | X |  |  |  |  |  |
|  | <14 months |  |  |  | X |  |  |  |  |  |  |  |  |  |  |  |  |  |  |  |  |  |  |  |  |  |  |  |  |  |  |  |  |
|  | 18 months |  |  |  |  |  |  |  |  |  |  | X |  |  |  |  |  |  |  |  |  |  | X |  |  | X |  |  |  |  |  |  | X |
|  | <2 years | X |  |  |  | X | X |  |  |  |  |  | X |  | X |  |  |  | X |  |  |  |  | X | X |  |  |  |  |  |  |  |  |
|  | 3 year |  | X |  |  |  |  |  |  |  |  |  |  |  |  |  |  |  |  |  |  |  |  |  |  |  |  |  | X | X |  | X |  |
|  | 4 year |  |  |  |  |  |  |  |  |  |  |  |  |  |  |  |  |  |  |  |  |  |  |  |  |  |  |  |  |  | X |  |  |
