# Supplementary material for: The evidence for services to avoid or delay residential aged care admission: a systematic review
Source: BMC Geriatr. 2019 Aug 8;19:217. doi: 10.1186/s12877-019-1210-3 (PMC6686247; doi:10.1186/s12877-019-1210-3)
Supplement: Supplementary file 3 — Additional forest plots. (DOCX 266 kb) [file 12877_2019_1210_MOESM3_ESM.docx]

# Additional File 3: Additional meta-analysis results in Forest Plots

# Figure S1 All interventions compared to control – mortality outcomes


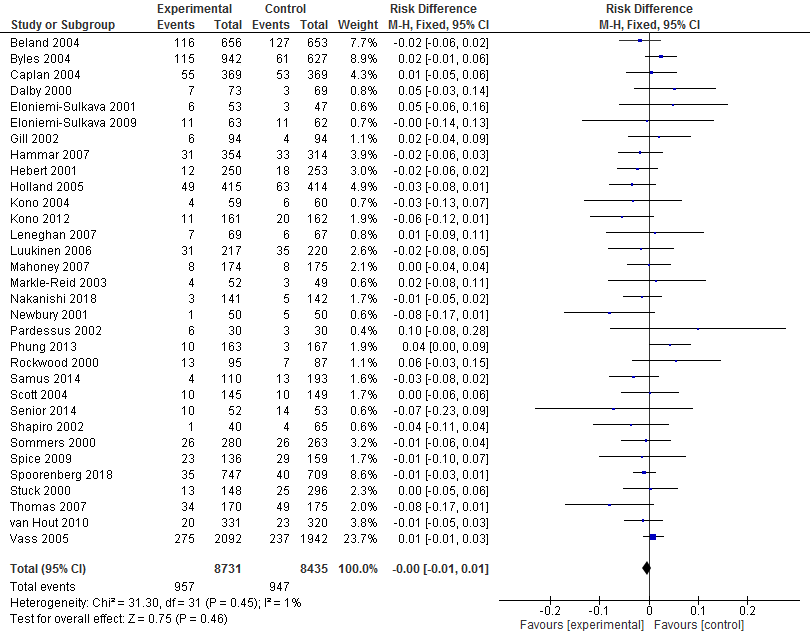


# Figure S2 All interventions compared to control – quality of life


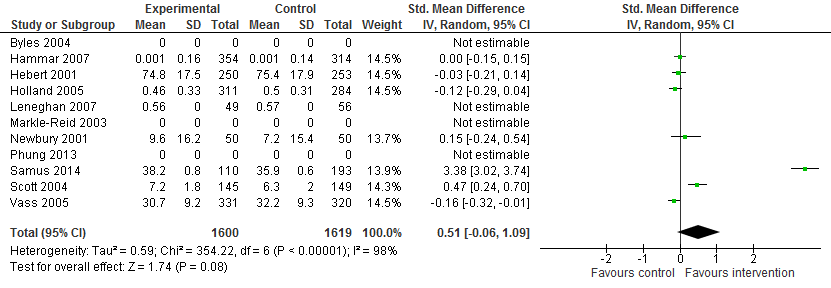


Figure S4 Dementia specific interventions compared to control **–** mortality


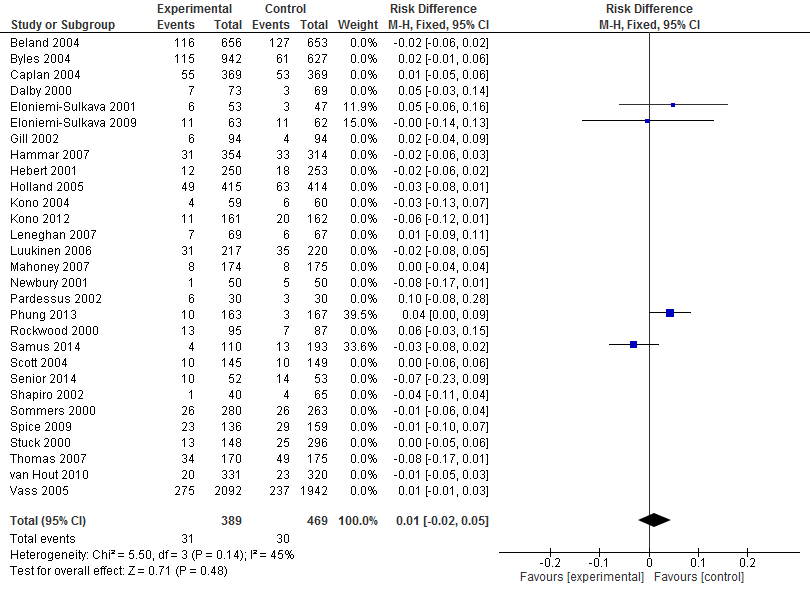


Note studies with 0% are not dementia specific interventions.

# Figure S5 Dementia specific interventions compared to control – quality of life


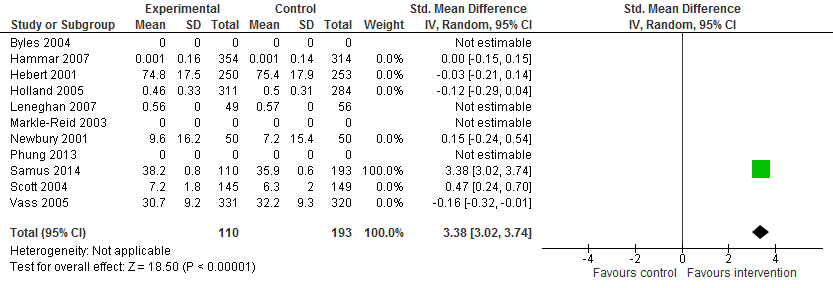


Note studies with 0% are not dementia specific interventions.

# Figure S6 Restorative interventions compared to control – RAC admissions


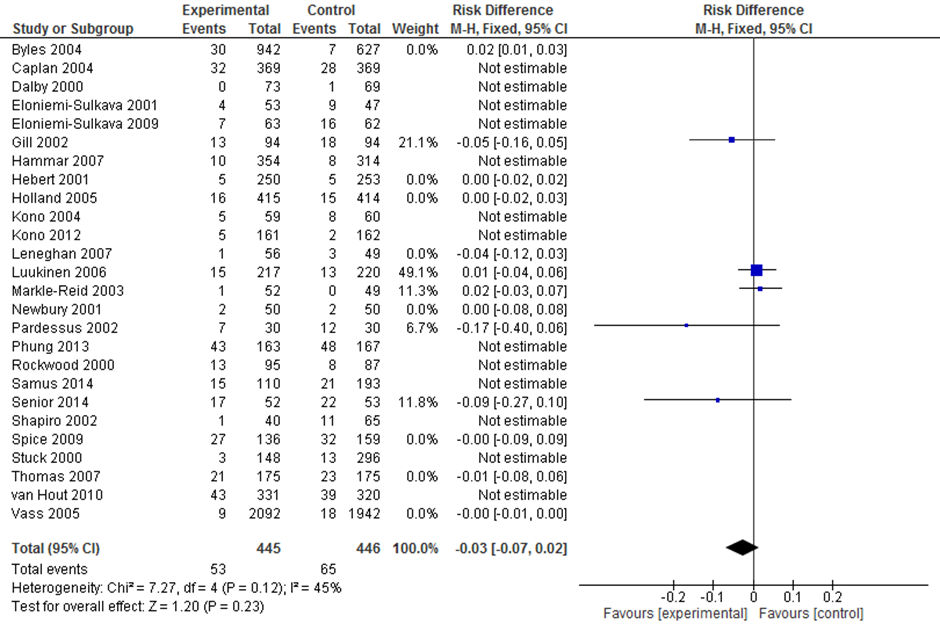


Note studies with 0% are not restorative interventions.
